# Implication of the anterior commissure in the allocation of attention to action

**DOI:** 10.3389/fpsyg.2014.00432

**Published:** 2014-05-19

**Authors:** Taylor J. Winter, Elizabeth A. Franz

**Affiliations:** Action, Brain, and Cognition Laboratory and fMRIOtago, Division of Science, Department of Psychology, University of OtagoDunedin, New Zealand

**Keywords:** attention, action system, anterior commissure, corpus callosum, goal-directed action

## Abstract

Our recent target article on the allocation of attention to action (herein called the AAA model; Franz, 2012) considered implicated subcortical processes and networks in people with intact corpus callosum (CC) and people without a CC due to commissurotomy or callosotomy. However, a small error in print—namely that the term “commissurotomy” was printed in place of “callosotomy” in some instances—led us to further explore whether any key functional roles have been attributed to the two primary cortical commissures (the anterior and posterior commissures) which remain intact in people with callosotomy, and if so, whether those would be relevant to our current AAA framework. Although existing evidence is sparse, here we consider the hypothesis that the anterior commissure (AC) is a remnant fiber tract which has been largely replaced with evolution of the CC (and we do not herein discuss the posterior commissure further). Indeed, a dearth of studies is available on the AC, calling the need for further research. Herein, we briefly review literature on the AC in humans and then propose a method that might be worthwhile to pursue in future studies.

The corpus callosum (CC) is the primary commissure connecting the two cerebral hemispheres of the brain. Therefore, while the term commissurotomy is not incorrect to describe the so-called “split-brain,” and is strictly correct for the original human surgical operations in which all commissures were severed (Gazzaniga et al., 1967), later surgeries severed only the CC, thereby bringing into use the term “callosotomy” which remains as the more appropriate term in those cases (e.g., Franz et al., 1996, 2000).

From an evolutionary perspective, the CC is a recent development observed only in placental mammals (Katz et al., 1983; Mihrshahi, 2006). The functionality provided by the CC is thought to be preceded, at least in part, by the anterior commissure (AC), as observed in non-placental mammals such as marsupials (Heath and Jones, 1971; Ashwell et al., 1996). Although some residual pathways of the AC are still present in the human brain, its connectivity and structure remains understudied in current literature and the sparse findings are rather inconsistent (Lasco et al., 2002; Patel et al., 2010; Choi et al., 2011). For example, findings based on magnetic resonance imaging (MRI) are mixed as to whether the AC is of similar size in females and males (Lasco et al., 2002; Patel et al., 2010). In a relatively recent study using the *in-vivo* technique of diffusion tensor imaging (DTI) which capitalizes on the diffusion of water molecules in axons, Patel et al. (2010) studied eight subjects and successfully isolated fibers of the AC in five subjects. Among the findings were, in one subject a large proportion of fibers in the posterior limb of the AC traveling bilaterally into parietal regions where most of them terminated, with a smaller bundle terminating in the precentral gyrus, and yet another bundle entering the temporal lobe. Such individual differences were found across all participants yet a majority of subjects showed bilateral projections to the orbitofrontal, parietal, and temporal cortices, of which the CC does not clearly project to. Together, the findings imply that the AC has both primary and vestigial projections to neocortical areas where the CC does not project, as others have also suggested (Di Virgilio et al., 1999; Patel et al., 2010).

In our view, the AAA relies heavily on neocortical networks and connectivity with subcortical structures such as the basal ganglia and thalamus (among others not discussed specifically in our proposal) (Franz, 2012). Thus, any evolutionary developments involving the AC might also have some role in the AAA model. However, we found little literature specifically addressing this possible link.

The role of the AC has been studied in tasks which are likely to require forms of attention, such as those involving visual attention. For example, it is well-known that acallosal populations have been studied in the context of tasks involving visual integration (Gazzaniga et al., 1962, 1965; Sperry, 1968; Levy et al., 1972). More recently, Corballis (1995) sought to address visual integration and attention in an effort to explain prior discrepancies in acallosal research. Corballis proposed a dichotomous visual attention system; one part (involving form) is automatic and localized to each hemisphere, and the other (involving movement and location) is voluntary and subcortical. The AAA model can build on this observation and address discrepancies observed in the literature. Based on our proposed model, across multiple levels of attention there are routes other than the CC that might mediate information transfer between hemispheres. One possibility involves the basal ganglia circuitry which we propose is potentially critical in mediating sharing of attention between hemispheres (Franz, 2012). By transecting the CC the direct transfer of attention via cortical pathways is largely abolished (unless, of course, the AC plays a backup role). Yet the attention-based integration of visual information could still be mediated via subcortical pathways and/or the AC. In this manner the two-part attention system observed by Corballis (1995) could be present due to the attention based mediation of visual integration via the AC (and/or the basal ganglia and involved circuits: Franz, 2012).

Studies involving vision and attention need not be considered entirely separately from the system regulating motor action. Based on our own findings (Franz, 2004; Franz and Packman, 2004), various forms of attention (visual and internal-nonvisual) are closely linked with motor action, and it stands to reason that a possible key to understanding a potential attention role of the AC lies in the link between action, vision, and attention. In an earlier study, Franz (2004) had normal participants draw circles bimanually under different manipulations of visual feedback, or internal attention to each hand. Size of the circles drawn by a hand receiving a form of internal attention became larger in a manner similar to effects that occurred with the presence of visual feedback of that hand (relative to no attention/visual feedback). Our AAA model implicates attention as being a key component in the regulation of motor output (as well as selection of what motor action is planned), and insofar as present evidence, we cannot rule out the AC as performing a role in that function. However, direct test of the role of the AC in any cognitive task is not trivial.

Testing whether the AC takes part in cognitive function is nearly impossible in neuropsychological research given that participants with localized AC are not easy to find (and we know of none). It might therefore be far more intuitive to assess neurological differences in the normal AC and correlate neurological data with behavioral data. The presented tractography method (elucidating the AC) can be applied relatively easily across large samples of the normal population, and ideally, can be combined with performance (based on tasks conducted during separate testing sessions from the DTI collection) of those same individuals using purpose-designed cognitive tasks. Herein, we demonstrate that DTI can be conducted and can yield useable results on a small sample of people from the neurologically-normal population. The general proposal would be to conduct similar methods on a larger sample size, and (as suggested above), correlate findings (of a proxy measure of the density of the AC, for example) with variables on a cognitive task.

In Figure 1 we show results of DTI tracking on data obtained from 10 female participants obtained from a public DTI dataset at Johns Hopkins University (see Acknowledgments for full source). Mean age was 22 years (*SD* = 2.5). Images were processed using FSL Diffusion Toolbox (Smith et al., 2004; Woolrich et al., 2009; Jenkinson et al., 2012) and procedures employed those used in previous studies on other fiber tracts (FDT; Behrens et al., 2003a,b, 2007; Johansen-Berg et al., 2004). Briefly, images were first concatenated into 4d volumes for each participant and skulls stripped using FSL's Brain Extraction Tool (BET; Smith, 2002; Jenkinson et al., 2005). Extracted images were corrected for minor head motion and eddy currents generated by changes in field direction. Diffusion tensors were fitted to generate images which display primary diffusion directions and levels of FA at each voxel to be used for color maps during masking. Bayesian estimation of diffusion parameters obtained using sampling techniques with crossing fibers (BEDPOSTX; “X” implying crossing fibers) was conducted to generate a distribution of diffusion size and direction accounting for two crossing fibers to later conduct tractography across diffusion voxels.

**Figure 1:**
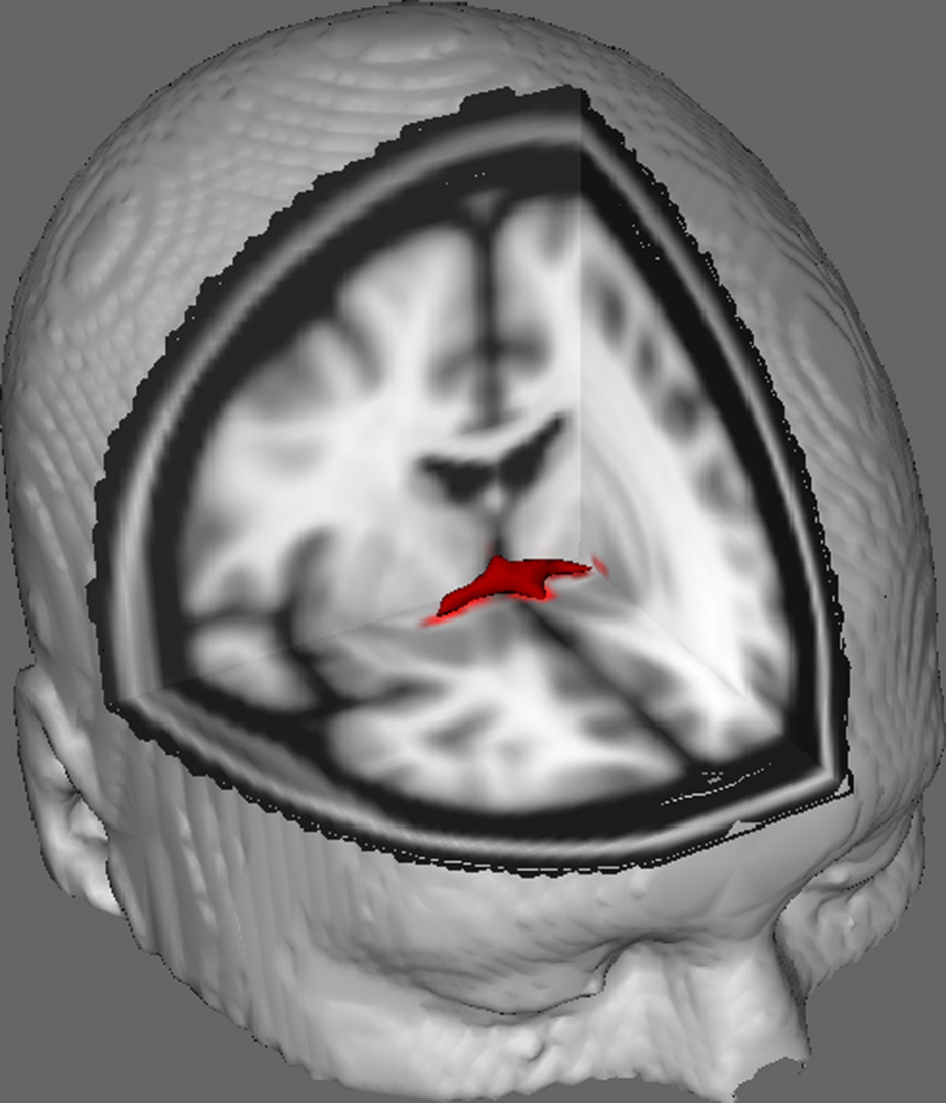
**Averaged tracking results across 10 control participants overlaid onto a standard T1-weighted MRI image with 2 mm isotropic voxels for anatomical reference**.

Averaged tracking results across all 10 control participants were then overlaid onto a standard T1-weighted MRI image with 2 mm isotropic voxels for anatomical reference. The group mean FA ratio was 0.3146 (*SD* = 0.0472). These tractography results reveal, importantly, that the structure of the AC can be elucidated, and therefore has potential for further studies correlating performance scores on behavioral tasks with FA values using a larger sample size.

In summary, at present, sparse literature exists, and of the available literature none specifically links the AC to processes related to the AAA model which is the focus of our earlier target article (Franz, 2012). Using a procedure similar to that proposed herein, it is possible to test larger sample sizes of the normal population, and correlate the FA values of the DTI results (for example) with a specific dependent variable obtained from a purpose-developed task of attention to action (procedures of which are an ongoing development in our laboratory).

## Conflict of interest statement

The authors declare that the research was conducted in the absence of any commercial or financial relationships that could be construed as a potential conflict of interest.
